# Mesenchymal stromal cell therapy for feline chronic gingivostomatitis: Long term experience

**DOI:** 10.3389/fvets.2023.1171922

**Published:** 2023-04-14

**Authors:** Maria Soltero-Rivera, Sterling Hart, Andrew Blandino, Natalia Vapniarsky, Boaz Arzi

**Affiliations:** ^1^Department of Surgical and Radiological Sciences, School of Veterinary Medicine, University of California, Davis, Davis, CA, United States; ^2^Department of Statistics, University of California, Davis, Davis, CA, United States; ^3^Department of Pathology, Microbiology, and Immunology, School of Veterinary Medicine, University of California, Davis, Davis, CA, United States; ^4^Veterinary Institute for Regenerative Cures, School of Veterinary Medicine, University of California, Davis, Davis, CA, United States

**Keywords:** gingivostomatitis, stromal cells, MSC, cats, dentistry, regenerative medicine

## Abstract

**Introduction:**

Mesenchymal stromal cells (MSC) therapy has emerged as a potential treatment option for refractory FCGS. However, there is a lack of long-term data on the use of MSC therapy in cats. This study aimed to evaluate the long-term safety and efficacy of MSC therapy for FCGS and investigate potential factors associated with treatment outcomes.

**Methods:**

This study was a retrospective evaluation of 38 client-owned cats with refractory FCGS who received MSC therapy. Medical records, histopathology, and the Stomatitis Activity Disease Index (SDAI) were reviewed. Correlations of the long-term follow-up success rates with SDAI and cell line type used were conducted. A client survey was also performed to assess side effect occurrence, quality-of-life following treatment, and overall treatment satisfaction.

**Results:**

Long-term follow-up ranged from 2 to 9 years post-MSC treatment. The overall positive response rate to MSC treatment was 65.5%, with 58.6% of cats exhibiting permanent improvement or cure. Adverse effects occurring during or immediately after treatment were noted in 34.2% of cases, the majority being transient, self-resolving transfusion-like reactions. No long-term adverse events were noted. No significant correlation in outcome was detected between allogeneic and autologous MSC treatment (*p* = 0.871) or the severity of the SDAI at entry (*p* = 0.848) or exit (*p* = 0.166), or the delta SDAI between entry and exit (*p* = 0.178). The status 6 months (none to partial improvement vs. substantial improvement to resolution) post-therapy was a predictor of long-term response (value of *p* < 0.041). Most clients were satisfied with the treatment and outcomes, with 90.6% willing to pursue treatment again, given a similar situation.

**Discussion:**

The results of this study support the use of both autologous and allogeneic MSC as an efficacious and safe therapeutic option for refractory FCGS.

## Introduction

Feline Chronic Gingivostomatitis (FCGS), a debilitating inflammatory oral mucosal disease, afflicts between 0.7 and 26% of the cat population to varying degrees (1–3). The disease is characterized by moderate to severe oral mucosal inflammation that clinically manifests as inappetence, lack of grooming, hypersalivation, bleeding from the mouth, lethargy, weight loss, and hyperemic mucosa. Histologically, the lesions are characterized by activated effector T and B cell infiltration of the oral mucosa, including CD4+ and CD8+ lymphocytes. There is also a notable systemic increase in effector CD8+ (cytotoxic) T cells with an associated reduction in central memory CD8+ cells (4–7). The complex pathogenesis of FCGS poses a challenge in forming a targeted treatment and prevention plan (8). Currently, the recommended treatment includes partial or full-mouth teeth extractions depending on the extent of inflammation present and evidence of concurrent periodontal disease and tooth resorption (9). Approximately 30% of patients are not responsive to extraction therapy and thus require continued use of immunosuppressors (i.e., corticosteroids, cyclosporine) or other immunomodulators (i.e., omega interferon) as well as pain medication and antibiotics (9). Patients that do not exhibit sufficient improvement (i.e., nonresponsive FCGS) are often euthanized due to poor quality of life (10). From an etiopathogenesis perspective, the current leading data suggests an ineffective immune response to chronic antigenic stimulation, likely feline calicivirus (FCV) infection (4, 11).

Mesenchymal stromal cells (MSC) modulate immune responses by reducing systemic levels of circulating T-cells, B-cells, natural killer cells, and dendritic cells, potentially making them ideal for immune-mediated inflammatory diseases like FCGS (12). Previously known as mesenchymal stem cells, after the 2005 issued clarification statement by The International Society for Cell & Gene Therapy (ISCT^®^) Mesenchymal Stromal Cell (ISCT MSC) committee, these are now recognized as mesenchymal stromal cells due to their secretory, immunomodulatory and homing properties (13). Recent data also support their use to shape a more effective antiviral immune response and lymphoid tissue regeneration in chronic viral infections (14, 15).

Systemically administered autologous and allogeneic adipose-derived MSC (adMSC) were first reported as a treatment for nonresponsive FCGS patients in 2015, and both cell types demonstrated marked improvement or complete resolution in most refractory patients (>60%), but discrepancies exist between the two (16, 17). Autologous administration offers practical benefits of a higher overall success rate (>70%) and shorter clinical response times as compared to allogenic adMSC (16, 17). However, the availability of allogeneic MSC, the possibility of banking and quality control, and the avoidance of deleterious effects of endogenous feline foamy virus (18) make this source more clinically feasible, pragmatic, and sustainable for practices integrating MSC into the array of treatment options. In concert, MSC treatment for refractory FCGS is a promising addition to clinicians’ treatment arsenal, with the choice between autologous vs. allogeneic administration requiring an educated decision based on a holistic overview of the patient’s condition and the administering facility’s capabilities.

The characterization of the positive outcomes following adMSC therapy in refractory FCGS cases provides evidence of a viable therapeutic option to traditional treatment methods. However, the long-term outcome of treatment efficacy and safety have not been comprehensively assessed to date. Therefore, we conducted a long-term retrospective study including 38 refractory FCGS patients with systemically-administered adMSC (Adipose-derived mesenchymal stromal cells; autologous and allogeneic) to better understand the long-term value of adMSC as a therapeutic option.

## Materials and methods

### Population

From 2013 to 2020, two clinical trials were conducted at the UC Davis Veterinary Medical Teaching Hospital, investigating the safety and efficacy of autologous and allogeneic MSC in treating nonresponsive FCGS cats. A total of 47 patients were selected for participation in this retrospective study. However, only 38 patients were ultimately included due to loss to long-term follow-up. All animal studies were conducted with the approval of the Institutional Animal Care and Use Committee and the Clinical Trial Review Board at the University of California, Davis. All owners signed informed consent. Eligibility criteria included cats affected by FCGS only, with no other comorbidities (i.e., FeLV, FIV, neoplasia), that did not respond to full-mouth extractions performed at least 6 months before enrollment. As described in the previously reported clinical trials, each cat received 20 million adMSC per treatment intravenously (IV) for a total of two treatments approximately 30 days apart (10, 11). Either autologous or allogeneic adMSC were administered. A final exit examination from the clinical trial was performed 6 months after the second treatment. Additional examinations were conducted on a case-by-case basis at later time points 18–108 months after the second treatment.

### Survey design and distribution

A client survey was designed to collect information on the extent of oral disease improvement (or lack thereof) years following treatment, duration of improvement, noticeable adverse events, medical history following the treatment, including diagnoses and prescribed medications, and overall satisfaction with treatment. The final survey included between 13 and 19 questions (Supplementary material). Variation in prompt number was due to the elimination of certain questions upon selection of previous answers. The latter allowed for an individualized, logical sequence with no unnecessary prompts. At this time, consent to obtain medical records from the patient’s primary care veterinarian was also requested. The survey was distributed *via* email through the survey software Qualtrics^®^ (Qualtrics XM)^1^. For owners who needed assistance completing this, the option was given to complete *via* phone conversation with one of the authors (SH), and the answers were directly inputted to Qualtrics.

### Primary care data collection

Based on information collected in the initial survey, participants who indicated the development of systemic or neoplastic disease, adverse events, or the recurrence of stomatitis were selected for further data collection from their primary care veterinarian. If present, the following history was collected: incidents of acute illness (i.e., hypersensitivity reactions, vomiting, diarrhea, respiratory distress, lethargy), related blood work changes (i.e., anemia, changes in renal or hepatic values, abnormalities in white blood cell counts, diabetes indicators), official diagnoses of the diseases, and cause of death (if applicable). Only medical developments following adMSC therapy were considered applicable to this study.

### Adverse events

To consider long term-adverse event, we identified cats who developed systemic diseases or other chronic conditions after the initial re-evaluation following treatment at 6 months. We then determined the rate of occurrence of the specific condition in the study population compared with the rate of occurrence in the general hospital population. The severity level of adverse events was determined using a standardized ranking process based on the NIH Adverse Event and Serious Adverse Event Guidelines, as defined in Table 1. We also recorded the rate of occurrence of short-term adverse events that may have occurred transiently, during, or immediately after treatment.

**Table 1:** The standardized scoring system used for evaluation of adverse effect severity level.

Severity level	Definition
Mild	Acute physical symptoms are observed or measured but have no observable discomforting behavioral effects on the patient. Veterinary assessment may be sought, but is unlikely to require outpatient treatment. Symptoms do not require hospitalization or cause long-term impairment, and resolve in a time period typical for the affliction.
Moderate	Acute physical symptoms are observed or measured and have minimal observable discomforting behavioral effects on the patient’s behavior. Veterinary assessment and/or outpatient treatment may be sought, but recommended treatments are minimal. Symptoms do not require hospitalization or cause long-term impairment and resolve in a time period typical for the affliction.
Severe	Acute physical symptoms are observed or measured and have moderate observable discomforting effects on the patient’s behavior. Veterinary assessment is strongly recommended, and outpatient treatment is likely necessary. Symptoms do not require hospitalization or cause long-term impairment and resolve in a time period typical for the affliction.
Serious	Acute physical symptoms are observed or measured in conjunction with severe observable discomfort in the patient. Immediate and/or continuous veterinary assistance is required. Serious adverse effects include scenarios that may result in the following: Death Near death Chronic or significant disability, incapacity, or disease Inpatient hospitalization or prolonged existing hospitalization

### Statistical analysis

Descriptive statistics were used to analyze the overall rate and extent of oral inflammation improvement, client satisfaction, and acute and possible long-term adverse events. For cats that exhibited a positive response to MSC therapy, the average period in which improvement persisted (days, weeks, months, years, or indefinitely) was calculated.

We applied a logistic regression model to explore the association between a patient’s long-term treatment outcome against other diagnostic variables of interest, including cell line type (autologous vs. allogeneic), SDAI scores (entry, exit, and change between entry and exit; Supplementary material), the patient’s status after 6 months (none to partial improvement vs. substantial improvement to resolution), and time to diagnosis (in months), while adjusting for age (years). For some covariates, a non-trivial amount of data was missing/unavailable, which, if excluded by following a complete-case analysis (CC), could lead to biased results by creating a subset of only “complete cases.” To account for this, we employed multiple imputations (MI) using the MICE package in R (13) that predicts missing values multiple times to account for the uncertainty with prediction. Model estimates and *p*-values were calculated for multiply imputed data *via* MICE and the complete-case data for comparison. *P*-values were considered significant when <0.05 and 95% confidence intervals were reached. All analysis was carried out by one of the authors (AB) in R statistical software [R Core Team (2022). R: A language and environment for statistical computing. R Foundation for Statistical Computing, Vienna, Austria].^2^

## Results

### Participant characteristics

Thirty-eight patients were identified for follow-up, receiving at least two doses of adMSC. Of those, 21 cats received autologous adMSC, and 17 received allogeneic adMSC. The mean follow-up period was 5.5 years (range 2–9 years). Patient ages at the follow-up time ranged from 6 to 15 years, with 16 patients declared deceased at the time of survey administration. Of the 38 patient’s families contacted, 29 completed the distributed survey *via* email or facilitated by phone correspondence. Seven surviving patients completed the survey and expressed willingness for an “in person” follow-up visit at UC Davis Veterinary Medical Teaching Hospital (VMTH) for re-evaluation and long-term SDAI scoring (Supplementary material).

### Clinical outcomes

**Figure 1:**
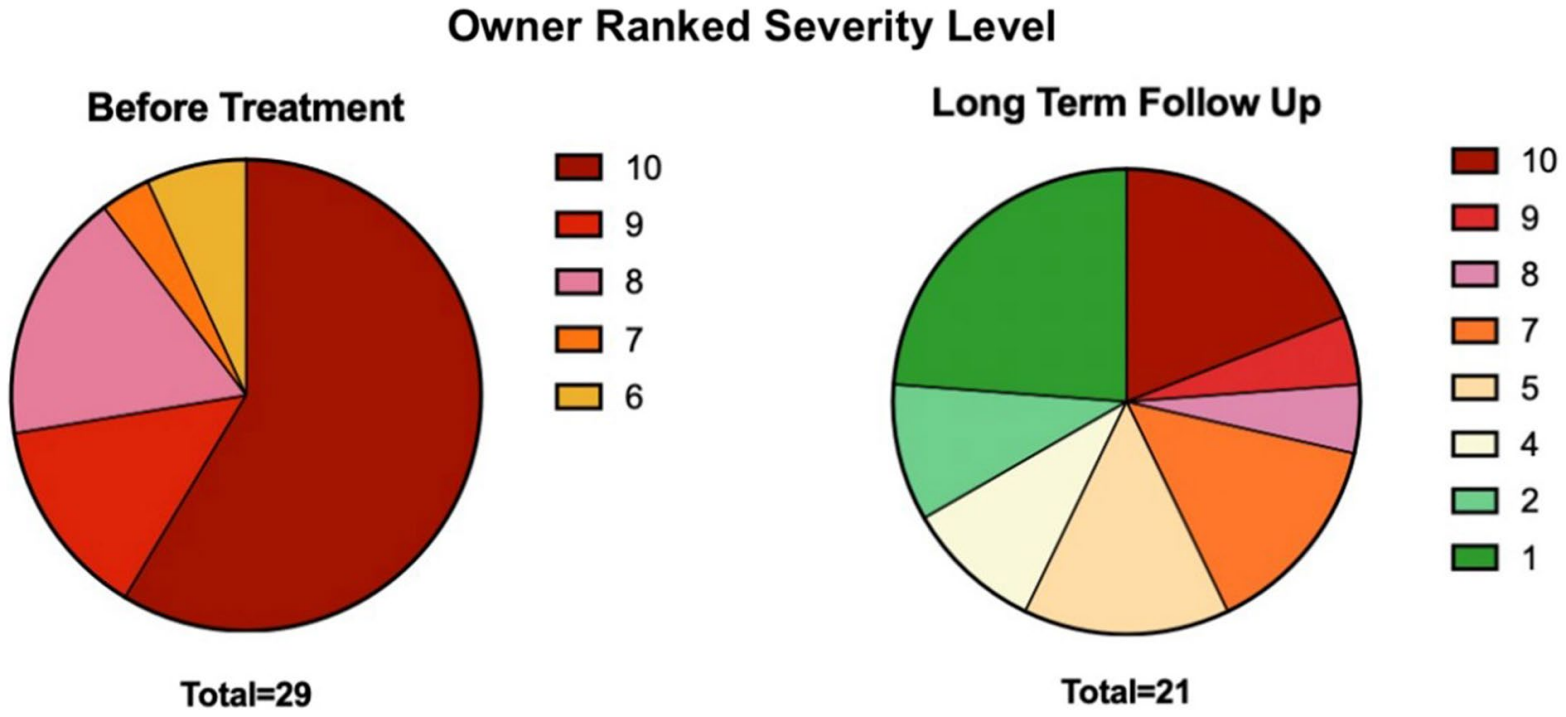
Distribution of severity levels before and after MSC treatment, subjectively scored by owners through the distributed survey. See Supplementary material questions 3–4 for the severity scoring prompt.

Owners classified patient disease severity levels before and after treatment on a scale of 1–10 (Supplementary material, Q3-Q4). Before treatment, all patients were classified at a severity level of 6 or higher, with a severity level of 10 comprising 58.6% of the population. Duration of long-term follow-up ranged from 2 to 9 years after receiving their first treatment. After treatment, a severity level of 10 was only seen in 19.0% of patients, and over half (56.8%) had a severity level between 1 and 5 (eight participants opted out of this question) (Figure 1).

Based on owner-ranked disease severity before treatment and at the time of follow-up, the positive response rate to adMSC therapy for all participants was 65.5% (19/29). Owners of patients receiving autologous adMSC perceived a 71.4% (10/14) positive response rate, whereas allogeneic adMSC recipients showed a 60.0% (9/15) owner-perceived positive response rate.

Owners were then prompted to define the permanence of improvement, or lack thereof (Supplementary material, Q5). Cats with a positive response following adMSC therapy until the time of follow-up or death were considered cases of permanent improvement, which comprised 58.6% of the study population (17/29 total, 9/17 autologous, 8/17 allogeneic). Cases of transient improvement, who experienced improvement and subsequent relapse of clinical signs, were seen in 17.2% of the study population (5/29 total, 3/5 autologous, 2/5 allogeneic). Of these five patients, three showed no improvement, and two showed partial improvement of the inflammation of the caudal oral cavity on gross oral examination during the trial’s exit visit (i.e., 6 months post-second adMSC administration). Twenty-four percent of cats (7/29 total, 2/7 autologous, 5/7 allogeneic) experienced no improvement at any time following treatment.

**Figure 2:**
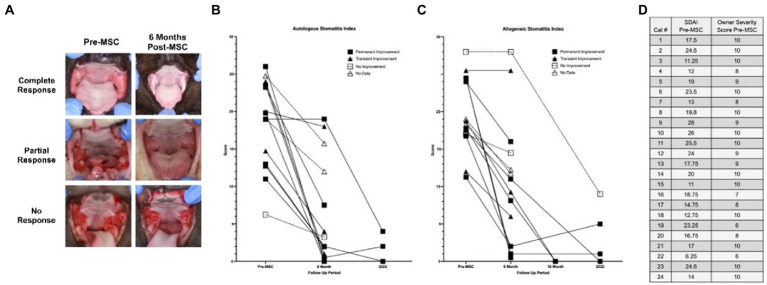
Long-term clinical follow up to measure disease severity. **(A)** Images obtained in original studies (Pre-MSC, 6 month follow-up) for three cases (complete response, partial response, and no response). **(B,C)** Stomatitis disease index (SDAI) of all patients having received allogeneic and autologous adMSC for all time points available (Pre-MSC, 6 months, 18 months, 2022-recheck). **(D)** Owner ranked severity level (1–10) compared with veterinarian-generated SDAI score (0–30; Supplementary material). SDAI scores were formed using a 0–3 scoring scale for ulceration, erythema, and/or proliferation of seven oral areas (maxillary attached gingiva, maxillary buccal mucosa, mandibular attached gingiva, mandibular buccal mucosa, palatoglossal arch, sublingual, molar gland), with an owner subjective ranking and weight change score added thereafter, also using a 0–3 scale.

SDAI scoring for the seven cats seen for long-term follow-ups is available in Figure 2. All cats experienced a reduction in SDAI relative to entry values with a mean of 83% reduction (range 67–100%). Nearly all exhibited a further reduction in SDAI relative to six-month exit values, with a slight increase in two cats. Interestingly, before treatment, in patients for which owners indicated a severity of 10, SDAI values varied between 11 and 26.

The logistic regression model investigating the correlation between long-term response with SDAI score at entry and delta-SDAI (SDAI entry-SDAI exit), cell line type, and time to diagnosis produced statistically insignificant results for both the multiple imputations (MI) model and linear regression analysis of complete cases (CC) (value of *p* = 0.85, 0.17, 0.87, 0.68, respectively). The logistic regression model produced a weak statistically significant correlation for larger SDAI exit scores with odds to long-term response for CC (value of *p* = 0.08), point estimate = 0.894 [95% CI-0.772, 1] for CC. The multiple imputation model for this variable produced statistically insignificant results (value of *p* = 0.166). Status at 6 months (none to partial improvement vs. substantial improvement to resolution) was a predictor of long-term response (value of *p* < 0.041 for both MI and CC), point estimate = 12.766 [95% CI-1.124, 144.998] for MI, 13.798 [95% CI-1.851, 291.672] for CC. Therefore, odds of positive long-term response for cats with substantial or complete improvement at 6 months positively correlated with their response to treatment.

### Safety

Adverse events occurring during or immediately after treatment were seen in 34.2% of cases, with a transfusion-like reaction being the most common (46%). Transfusion-like reactions included one or more of the following clinical signs during or within hours of treatment: vomiting (5/6), diarrhea (2/6) and increased respiratory rate (2/6). Sixty-seven percent of transfusion-like adverse events occurred during autologous adMSC administration and 33% during allogeneic adMSC administration. Other adverse events included lethargy (7.7%, 1/13), mild respiratory rate increase (38.4%, 5/13), and one incident of vomiting 12 h post-treatment. Between autologous and allogeneic adMSC and considering the time during administration up to 12 h post-treatment (except one patient experiencing lethargy 4 days post-treatment), autologous administration had a higher overall rate of adverse events occurring in 69.2% as compared to the 30.8% seen with allogeneic treatment.

The severity level of adverse events, classified based on the scale defined in the materials and methods, demonstrated that 61.5% (8/13) of events were considered mild, 15.4% (2/13) considered moderate, and the remaining 23.1% (3/13) classified as severe. The severe adverse events characterized by pronounced transfusion-like reactions occurred in three cats. Two of these patients experienced nausea and/or vomiting, diarrhea, and respiratory distress during treatment. The latter symptoms required immediate medical intervention and cessation of adMSC administration. The third patient became obtunded, had respiratory distress, and urination immediately following adMSC administration. These clinical signs resolved within 10–15 min of cessation of adMSC administration. There were no incidents of death during or immediately after adMSC administration.

Records provided by primary care providers were moderately inconsistent, with gaps in time and ambiguity. Nevertheless, a tally of all recorded diagnoses and the time between adMSC treatment until diagnosis was made are available in Table 2.

**Table 2:** Occurrence of disease following treatment in the study population.

Affliction	Total observed occurrences	Percentage value	Average time of onset (days)	Prevalence in normal population
Anemia	3	7.9% (3/38)	138 (Range 98–213)	3.6%
Hyperthyroidism	2	5.30% (2/38)	1,583 (Range 1,052–2,144)	13.9%
Renal disease	4	10.5% (4/38)	1023.3 (Range 98–2,115)	23.1%
Cardiac disease	3	7.90% (3/38)	523 (Range 45–1,303)	4.3%
Gastrointestinal disease	1	2.6% (1/38)	2,464	2.0%
Neoplasia	4	12.20% (4/38)	759.8 (Range 204–2,115)	0.5–37.7%

The average onset is from the time of treatment to the first record of diagnosis. If a particular disease is not included, assume there were no observed occurrences. Normal population statistics are provided for reference (19–22).

### Client satisfaction

From phone and email surveys conducted, overall client satisfaction was positive, with 90.6% of clients willing to pursue treatment again if a similar situation arose, 6.3% not willing to pursue treatment again, and 3.1% unsure (Supplementary material, Q11).

Owners were then prompted to rank factors influencing their willingness to pursue treatment again. Ninety percent of owners ranked the degree of improvement in the pet’s illness as important or very important. The availability of other treatment options and their success rates were considered important by 71% of owners. The ease of administering oral medication and the number of required recheck appointments were ranked as important by 52 and 22% of participants, respectively (Supplementary material, Q13). Clients were then prompted to write in other factors that were important to them personally, which brought cost, distance to the treatment facility, potential side effects, and pet comfort level during treatment into the discussion.

## Discussion

This is the first study to investigate the long-term safety and efficacy of adMSC for the treatment of refractory FCGS. This retrospective study demonstrates several clinically important findings. First, in most cases, systemically administered autologous and allogeneic adMSC produce marked, permanent improvement. Second, the clinical success rate of autologous adMSC was higher than allogeneic cells but not significantly. Third, adMSC therapy was safe with no long-term adverse events. However, transfusion-like reactions were not uncommon, especially in participants receiving autologous adMSC. Additionally, a patient’s clinical response at 6 months post-treatment predicts the long-term positive response to adMSC therapy. Finally, client satisfaction with adMSC therapy for refractory FCGS was vastly positive.

Our study population’s overall positive response rate was 65.5%, based on owner-ranked disease severity before and after treatment. All patients that received oral exams at long-term examination exhibited a reduction in SDAI compared to entry values, and nearly all exhibited a further reduction relative to the six-month exit score, except for two patients. Overall, a mean 83% reduction in SDAI was observed. In addition, in 58.6% of cases, improvement was long-lasting (36–108 months). The long-lasting positive response rate allows for greater confidence in treatment efficacy than the initial response rate alone. This study’s recruitment percentage agreed with the reported 30% of non-responders to traditional full-mouth tooth extractions (9, 23). Hence, we postulate that the 65.5% overall success of adMSC may reduce the number of patients classified as refractory from 30% to just 10.3% and concurrently may increase the overall rate of remission for FCGS from ~70% (9, 23) to ~90%.

We observed a difference in clinical success rate between autologous and allogeneic adMSC, as was noted in previous studies, with long-term autologous success at 71.4% and allogeneic at 60.0%. Specifically, the success rate was previously reported to be 71% in autologous and 57% in allogeneic treatment groups in the early original short-term studies (16, 17). The slight variations between the success rates of the cell types may result from this study’s owner subjective response rankings, the considerable increase in participant group size, or the longer follow-up period. In the case of allogeneic adMSC recipients, authors originally found a delayed response rate as compared to autologous cells (16), which may explain this study’s observed slight increase in overall allogeneic response rate over a longer period. Lastly, the reason for allogenic adMSC underperforming relative to autologous is unclear and warrants further investigation.

Most adverse events noted were characterized by mild to moderate transfusion-like reactions, all of which resolved promptly. In these patients, subsequent treatments were administered at a slower rate, eliminating the occurrence of adverse reactions. The incidence of transfusion-like reactions was not unexpected (24). However, the occurrence of these reactions in autologous vs. allogeneic cell lines was different. Host-derived autologous cells are expected to carry less risk of reaction, but we observed two times more transfusion reactions in autologous treatments than in allogeneic. According to the clinicians’ observations, a faster rate of adMSC administration was associated with transfusion-like reactions. Because the first clinical trial was solely investigating autologous cells, most transfusion-like reactions were noted at these early stages and are likely to be due to lack of knowledge and experience in needing to administer adMSC at a slow pace, i.e., over 30 min.

Establishing indicators of patient likelihood to respond to MSC treatment is desirable in implementing effective, personalized treatment. Our study found that a positive clinical response 6 months post-treatment positively predicted the odds for a long-term response. While this does not offer a prediction of response before designing a treatment plan, this may be useful for clinicians advising owners on the treatment’s future success and determining whether to pursue additional MSC or alternative treatments. Therefore, 6 months should be used as a standard checkpoint for clinicians in determining whether the treatment has been or will continue to be successful to avoid premature conclusions on patient response.

The vast majority of clients responded positively when asked if they would pursue a similar treatment for their cat in the future. Client satisfaction and patient health improvement demonstrate the potential for adMSC therapy to become in high demand for other pet morbidities in veterinary practices.

Due to missing data in the logistic regression models, the current analyses may not reflect accurately evaluated correlations. Further prospective studies with larger sample sizes may reveal one or more of these variables as useful markers in designing patient treatment plans, solidify the results of this study, and may help improve the determination of the timing of treatment to optimize outcomes. A lack of substantial, continuous data from primary care veterinarians prevented a confirmed analysis of long-term disease occurrence in the study population. With this in mind, the results obtained were compared with the general hospital population, demonstrating a higher prevalence of anemia, cardiac disease, and a slight increase in gastrointestinal disease in the study population. Higher than normal prevalence of anemia in the study population was not unexpected as all cats included in the study suffer from chronic disease (25). Likewise, though unknown, an association between oral disease with cardiac and gastrointestinal dysfunction cannot be ruled out as a causative factor for the increased disease rate of these systems seen in the study population. Interestingly, FCGS was associated with a high incidence of esophagitis, most likely owing to the altered microbiome of the oral cavity (26). Similar effects may contribute to the gastrointestinal disease seen in cats evaluated in this study. However, other differential diagnosis for infiltrative gastrointestinal disease, such as inflammatory bowel disease or lymphoma, cannot be completely ruled out. Provided a more complete data set, disease incidence rates may shift in either direction from those found.

The SDAI used in this (Supplementary material) and previous studies (27) has been a valuable tool for patient diagnosis and status monitoring, but certain aspects of the scoring process may alter the results. This study found only a weak association between SDAI exit scores and outcomes. A study on a larger population would be better for determining if the odds of a positive response decrease by a certain percentage for each 1-unit increase in the patient’s SDAI exit score. As shown in Figure 2D, disparity exists between the owner and clinician assessment of patient disease status, both of which are necessary for generating an SDAI score. With these two values at odds, scores may not truly reflect patient status; therefore, a more objective scoring system should be developed, or the SDAI should focus on either the client or the clinician evaluation (i.e., two separate SDAIs). As described in previous studies, elevated CD8+ T cell levels, interferon-γ, and interleukin (IL)-1β concentrations, blood neutrophilia, hypergammaglobulinemia, abnormal CD4/CD8 ratios, as well as calicivirus and foamy virus positivity are associated with FCGS severity and response to therapy (16). Developing a scoring system that integrates these factors in the assessment of disease severity may create a more comprehensive value for use in a clinical setting. Furthermore, the use of these biomarkers in the initial assessment of patients may be used in the future in creating personalized treatment plans based on the patient’s disease status. In the past, refractory patients unresponsive to extraction therapy would pursue supplementary MSC transfusion months later. It is possible that MSC treatment immediately after extraction therapy may be more beneficial, a trend that is currently being explored in veterinary and human practice.

In conclusion, this long-term retrospective study demonstrates the safety and efficacy of both autologous and allogeneic adMSC and provides valuable information for patients and clinicians. The long-term success exhibited by most patients, regardless of cell type, is encouraging. FCGS-affected cats refractory to tooth extraction therapy may benefit from adMSC interventions.

## Data availability statement

The original contributions presented in the study are included in the article/Supplementary material, further inquiries can be directed to the corresponding author.

## Ethics statement

The animal study was reviewed and approved by the IACUC. Written informed consent was obtained from the owners for the participation of their animals in this study.

## Author contributions

MS-R: study concept and design, evaluation of data, drafting of the manuscript, and principal investigator. SH: acquisition of data, evaluation of data, and drafting of the manuscript. AB: statistical analysis, evaluation of data, and revision of the manuscript. NV: evaluation of data and revision of the manuscript for important intellectual input. BA: study concept and design, evaluation of data, and drafting of the manuscript. All authors contributed to the article and approved the submitted version.

## Funding

Financial support for the clinical trials was provided by the NIH 1R21DE024711-01, the WINN Feline Foundation’s Miller Trust grant, and the George and Phyllis Miller Feline Health Trust of the San Francisco Foundation and administered by the Center of Companion Animal Health, UCD.

## Conflict of interest

The authors declare that the research was conducted in the absence of any commercial or financial relationships that could be construed as a potential conflict of interest.

## Publisher’s note

All claims expressed in this article are solely those of the authors and do not necessarily represent those of their affiliated organizations, or those of the publisher, the editors and the reviewers. Any product that may be evaluated in this article, or claim that may be made by its manufacturer, is not guaranteed or endorsed by the publisher.

## Footnotes

^1^ www.qualtrics.com

^2^ http://www.R-project.org/

## Abbreviations

FCGS: Feline chronic gingivostomatitis
adMSC: Adipose-derived mesenchymal stromal cells
MSC: Mesenchymal stromal cells
FCV: Feline calicivirus
SDAI: Stomatitis disease activity index
