# Supplementary material for: Mesenchymal stromal cell therapy for feline chronic gingivostomatitis: Long term experience
Source: Front Vet Sci. 2023 Apr 14;10:1171922. doi: 10.3389/fvets.2023.1171922 (PMC10165997; doi:10.3389/fvets.2023.1171922)
Supplement: Supplementary file 1 [file Data_Sheet_1.docx]

***Supplementary Material***

**Supplementary Figure 1.** Final survey distributed to owners via email. Participants opting for phone surveys were presented with verbatim prompts, in the same adaptive order as they would appear in online format.

Q1

How old is [Pet’s Name] today?

- Less than 5 years
- 5 years but less than 10
- 10 years but less than 16
- 16 but less than 20
- 20 years or older
- My pet is deceased

Q2

Which of the following reasons prompted you to have [Pet’s Name] seen at the University of California-Davis Veterinary Medical Teaching Hospital (UCD-VMTH)? Please check ALL of the following that apply:

- Painful mouth (i.e., increased salivation, pawing at the mouth, preferential chewing on one side, vocalizing when eating, preference for soft food)
- Discharge or drainage from the oral cavity
- Poor response to previous treatments
- Halitosis (i.e., bad breath)
- Cat was less active
- Decreased or absent appetite
- Weight loss

Q3
Reflecting on [Pet’s Name]'s participation in the stem cell clinical trial, how would you rate the severity of [Pet’s Name]'s illness at the beginning of treatment?

(1 indicates a mild level and 10 indicates a severe level)

- Mild (1)
- (2)
- (3)
- (4)
- Moderate (5)
- (6)
- (7)
- (8)
- (9)
- Severe (10)
- No disease or illness present (0)
- Unsure (12)

Q4
How would you rate the severity of your [Pet’s Name]'s illness NOW or at the time of death?
(1 indicates a mild level and 10 indicates a severe level)

- Mild (1)
- (2)
- (3)
- (4)
- Moderate (5)
- (6)
- (7)
- (8)
- (9)
- Severe (10)
- Cured/Resolved (0)
- Unsure (12)

Q5
In your opinion, how permanent was [Pet’s Name]'s improvement after treatment at UC Davis? Please check ONE of the following:

- Permanent improvement (1)
- Temporary improvement for weeks/months/years (2)
- No improvement (3)

Q6
Did you notice any side effect(s) in [Pet’s Name] that you thought might have been due to the treatment administered? Please check ONE of the following:

- Yes (1)
- No (2)

Q7 *Display This Question If Q6 = Yes*

Please briefly describe the side effect(s).

Q8 *Display This Question If Q6 = Yes*

How soon after the treatment did the side effect(s) occur?

- Years (4) __________________________________________________
- Months (5) __________________________________________________
- Weeks (6) __________________________________________________
- Days (7) __________________________________________________

Q9 *Display This Question If Q6 = Yes*

For how long did the side effect(s) persist?

Q10 *Display This Question If Q6 = Yes*
How severe would you rate [Pet’s Name]'s side effect(s)?
 (1 indicates a mild level and 10 indicates a severe level)

- Mild (1)
- (2)
- (3)
- (4)
- Moderate (5)
- (6)
- (7)
- (8)
- (9)
- Severe (10)
- Cured/Resolved (0)
- Unsure (12)

Q11
Based upon your experience, if the same circumstances arose again, would you be willing to have [Pet’s Name] undergo a similar or identical treatment? Please check ONE of the following:

- Yes (1)
- No (2)
- Unsure (3)

Q12 *Display This Question If Q11 = Yes, No, Unsure*

Please briefly state the reason(s) why you [would/would not/are unsure if you would] be willing to have [Pet’s Name] undergo a similar or identical treatment?

Q13
How important would each of the following factors be in your willingness (or not) to have [Pet’s Name] treated in the same way again?

[Please rank each factor on a scale of: Not Important (1), (2), (3), (4), Moderately Important (5), (6), Important (7), (8), (9), Very Important (10), Not Applicable (11)]

- Degree of improvement in your cat’s illness (1)
- Other treatment options available and their success rates (i.e. interferon omega, cyclosporin, CO2 ablative laser therapy, chronic steroid therapy, chronic pain management) (2)
- Ease of giving oral medications (i.e. pills, liquid) (3)
- Number of recheck appointments (4)

Q14
Please briefly explain any other factor(s) that would influence your willingness (or not) to have [Pet’s Name]'s treated in the same way again.

Q15
Has [Pet’s Name] been treated or is currently under treatment for any of the following medical conditions? Please select all that apply.

- Diabetes mellitus (1)
- Kidney disease (2)
- Heart disease (3)
- Liver disease (4)
- Gastrointestinal ulceration (5)
- Vomiting (6)
- Anorexia (7)
- Diarrhea (8)
- Flare up of a viral infection (9)
- Toxoplasmosis (10)
- Eye disease (11)
- Cancer (12)
- My cat has not been treated for any of the above conditions (13)

Q16 *Display This Question if pet is not deceased*

Would you be willing to bring [Pet’s Name] to the UCD VMTH for a long-term awake follow-up evaluation of his/her oral cavity?

- Yes (1)
- No (2)

Q17

Do we have your permission to obtain [Pet’s Name]'s medical records from follow-up visits at your primary care veterinarian after stem cell therapy?

- Yes (1)
- No (2)

Q18 *Display This Question if pet is deceased*
Please accept our sincerest condolences on the loss of [Pet’s Name].

We are interested in knowing which of the following factor(s) caused the death of [Pet’s Name] or contributed to making the decision of putting [Pet’s Name] to sleep:

- My pet was euthanized due to poor quality of life related to oral disease (13)
- My pet was euthanized due to poor quality of life related to systemic disease (i.e., kidney disease, liver disease, cardiac disease) (14)
- My pet was euthanized due to poor quality of life related to cancer (15)
- My pet died due to unknown causes (17)
- My pet died of natural causes or due to progression of a systemic illness (18)
- My pet died due to severe trauma (20)
- Other (Please write in): (19)

Q19 *Display This Question if Q5 = Temporary improvement for weeks/months/years*

How long did the improvement last? Please indicate the number(s) of years, months, weeks, or days to the best of your ability.

- Years (4) __________________________________________________
- Months (6) __________________________________________________
- Weeks (7) __________________________________________________
- Days (8) __________________________________________________
