# Supplementary material for: Mesenchymal stromal cell therapy for feline chronic gingivostomatitis: Long term experience
Source: Front Vet Sci. 2023 Apr 14;10:1171922. doi: 10.3389/fvets.2023.1171922 (PMC10165997; doi:10.3389/fvets.2023.1171922)
Supplement: Supplementary file 2 [file Data_Sheet_2.doc]

STOMATITIS DISEASE ACTIVITY INDEX

PATIENT: ­_________________________________________________________

Date: __________ Weight today: ­­­­­­­­­­­­_________

DIET: ___________________________________________________________

Client Report: Please evaluate the following (circle one number for each of the following criteria):

**Appetite**: 3 = eats only pureed food, or only when hand fed 2 = eats wet food on his/her own; cannot eat dry food 1 = eating wet and dry food, but less than normal amount 0 = eating normally

**Activity level**: 3 = no interest in people or other pets, spends most of time sleeping 2 = low activity level, but will play occasionally when engaged by people or other pets 1 = plays spontaneously, but not frequently 0 = normal activity level (playful and active)

**Grooming behavior**: 3 = will not groom 2 = grooms occasionally but not at ‘pre-illness’ level 1 = grooming excessively 0 = grooming normally

**Perceived comfort**: On a scale of 0-3, with 0 being most comfortable and 3 being most painful, rank your cat’s present comfort level: ______

Clinician Evaluation:

| STOMATITIS DISEASE ACTIVITY INDEX | **0** | **1** | **2** | **3** |
| --- | --- | --- | --- | --- |
| Owner evaluation |  |  |  |  |
| Weight |  |  |  |  |
| Maxillary buccal mucosal inflammation |  |  |  |  |
| Mandibular buccal mucosal inflammation |  |  |  |  |
| Maxillary attached gingival inflammation |  |  |  |  |
| Mandibular attached gingival inflammation |  |  |  |  |
| Molar salivary gland inflammation |  |  |  |  |
| Inflammation of areas lateral to palatoglossal folds |  |  |  |  |
| Oropharyngeal inflammation |  |  |  |  |
| Lingual and/or sublingual inflammation |  |  |  |  |
| TOTAL SCORE (maximum = 30) | | | |  |

Owner evaluation: average of the circled values above

Weight: 0 = gain >0.5kg 1 = gain >0.25kg but <0.5kg 2 = <0.25kg gain 3 = weight loss (if lost >0.5kg compared with most recent visit)

Inflammation of oral cavity sites: 0 = none 1 = mild 2 = moderate 3 = severe
